# Kinetics and Chemorheological Analysis of Cross-Linking Reactions in Humins

**DOI:** 10.3390/polym11111804

**Published:** 2019-11-02

**Authors:** Anna Sangregorio, Nathanaël Guigo, Ed de Jong, Nicolas Sbirrazzuoli

**Affiliations:** 1 Avantium Chemicals B.V., Zekeringstraat 29, 1014 BV Amsterdam, The Netherlands; anna.sangregorio@avantium.com (A.S.); ed.dejong@avantium.com (E.d.J.); 2 Institut de Chimie de Nice, Université Côte d’Azur, CNRS, UMR 7272, 06108 Nice, France; nathanael.guigo@univ-cotedazur.fr

**Keywords:** lignocellulosic biomass valorization, humins, biobased thermoset, kinetics, isoconversional method

## Abstract

Humins is a biomass-derived material, co-product of the acid-catalyzed conversion of cellulose and hemicellulose to platform chemicals. This work presents a thorough study concerning the crosslinking kinetics of humins by chemorheological analysis and model-free kinetics under isothermal and non-isothermal curing. Humins can auto-crosslink under the effect of temperature, and the reaction can be fastener when adding an acidic initiator. Thus, the effect of P-Toluenesulfonic acid monohydrate (pTSA) on the crosslinking kinetics was also studied. The dependencies of the effective activation energy (*E*_α_-dependencies) were determined by an advanced isoconversional method and correlated with the variation of complex viscosity during curing. It is shown that humins curing involves multi-step complex reactions and that the use of an acidic initiator allows faster crosslinking at lower temperatures, involving lower *E*_α_. The shift from chemical to diffusion control was also estimated.

## 1. Introduction

The necessity to focus on biorefinery technologies is exemplified by the growing concern on climate change and the exhaustion of fossil raw materials. Valorization of lignocellulosic biomass is one of the most promising solutions to face the depletion of fossil feedstocks worldwide [1,2,3,4]. Humins is a biobased material, co-product of the acid-catalyzed conversions of cellulose and hemicellulose to platform chemicals. First studies concerning humins date almost one century [5], however only in the last few years scientists took an interest in the formation and the characterization of this product [6,7,8,9,10,11]. Sumerkii et al. [6] first postulated that humins formation occurs via 5-hydroxymethylfurfural (HMF) which goes through polycondensation processes by electrophilic substitution. Later, aldol addition and condensation were proposed as key reactions in the acid-catalyzed growth of humins, adding HMF to 2,5-dioxo-6-hydroxy-hexanal (DHH) [7]. Uncertainties are also related to humins chemical structure, which is strictly linked to the conditions under which it is formed. There exists a consensus that humins are a carbonaceous, heterogeneous, and polydisperse macromolecules with a furanic structure containing aldehydes, ketones, and hydroxyls as main functional groups [8,9,10,11,12,13,14,15]. It has been demonstrated that humins can be applied in a wide range of applications [14,15,16,17,18,19,20,21]. Lately, we demonstrated the potential of humins as thermosetting to produce all green composites [22] and for wood modification [23]. To further exploit the use of humins as thermosetting resin, the study of the curing kinetics plays a crucial role in developing and optimizing the industrial manufacturing process. Curing kinetics can be used to minimize the cycle time or together with models of chemorheology, heat transfer and micromechanics to calculate and minimize the generated internal stresses. In this way, it is possible to improve the process development and ultimately the quality and performance of the final product [24,25,26,27].

**Scheme 1:**
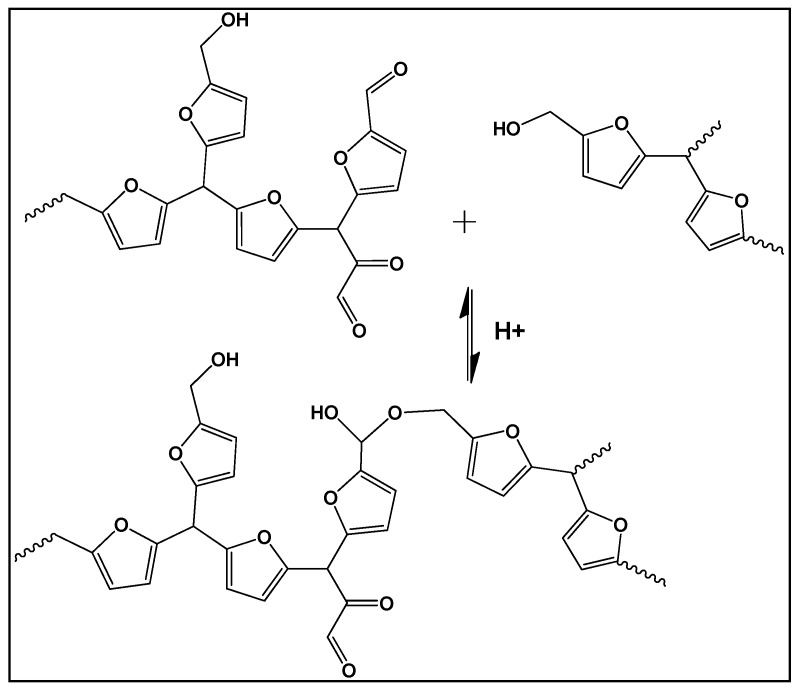
Acid-catalyzed hemiacetal formation within humins chains.

This study aims to understand the curing behavior of humins, highlighting the changes in mechanisms when undergoing auto-crosslinking (i.e., without a catalyst) and by using an acid catalyst. The chemorheological behavior of the curing systems was first studied by differential scanning calorimetry (DSC), and dynamic rheometry. The study was done for isothermal and non-isothermal data. Due to the complexity of the macromolecular system, it was decided to support isothermal data by Topem^®^ measurements, a multi-frequency temperature-modulated DSC technique, which involves stochastic modulation of temperature. This technique allows the evaluation of the quasi-static heat capacity (*c_p_*_0_). In the case of non-isothermal data, kinetic data from the storage modulus variation evaluated by dynamic rheology were obtained. All data were treated with an advanced isoconversional method to yield the dependence of activation energy on crosslinking conversion. This work reports the first chemorheological study concerning humins cross-linking. Scheme 1 highlights a postulated structure of humins based on previous works [8,9,10,13] and a potential acid-catalyzed hemiacetal formation between two humins chains. Such possibilities of cross-links among reactive groups allow formation of new all green thermosets, which hold great potential as building materials, medium-density fiberboard or in automotive applications. Humins structure suggests a very good affinity with natural lignocellulosic fibers, such as flax or jute, to produce all green composites by using a fully biobased resin [22].

## 2. Materials and Methods

### 2.1. Materials

Humins were supplied by Avantium N.V. and produced in their pilot plant in Geleen, The Netherlands, by conversion of fructose and glucose. Humins samples were used as received. P-Toluenesulfonic acid monohydrate (pTSA) was supplied by Sigma Aldrich, (Mw = 190.22 g/mol, melting point = 103–106 °C, purity > 98.5%). PTSA was chosen as a catalyst to study humins polymerization under acidic conditions. For this purpose, a sample was prepared by heating humins for 20 min at 80 °C and then 1.5 wt % of pTSA was added. The mixture was stirred for another 20 min at 80 °C to obtain a homogenous sample.

### 2.2. Differential Scanning Calorimetry (DSC)

A heat flux Mettler-Toledo DSC-1 was used to perform conventional differential scanning calorimetry (DSC) and stochastically temperature modulated differential scanning calorimetry (Topem^®^ by Mettler-Toledo, Columbus, OH, USA) measurements. Computations for the kinetics evaluation were performed by an internal software written by Sbirrazzuoli and regularly updated [28,29,30], able to treat any kind of isothermal and non-isothermal data. Temperature, enthalpy and tau lag calibrations were performed using indium and zinc standards. Around 7 mg of humins were placed in a 40 µL aluminum crucible and closed by a perforated pan lid. Isothermal measurements were performed on crude humins by standard DSC at 120, 140, and 160 °C. The same set of temperatures was used to perform quasi-isothermal measurements by Topem^®^ to evaluate the quasi-static heat capacity (*c*_p0_). The amplitude of the temperature perturbation was fixed at 0.5 °C for all the stochastically modulated experiments and the period of pulses was ranging from 15 to 30 s. 

### 2.3. Rheology

Viscosity, storage (*G*’) and loss (*G*’’) moduli of crude humins and humins/pTSA were registered with a Thermo Scientific HAAKE MARS rheometer. Measurements were obtained on oscillatory mode with plate-plate geometry (25 mm diameter and 1 mm gap). Experiments were carried out at non-isothermal conditions with a heating rate of 0.5, 1, 2 °C·min^−1^ on crude humins and humins/pTSA. Isothermal measurements were carried out on crude humins at the same temperatures as DSC and Topem^®^ experiments (120, 140, and 160 °C). For all measurements, a constant frequency of 1 Hz and amplitude equal to 0.05% was used to ensure being in the linear viscoelastic domain. Gelation time was evaluated as the crossing point of storage (*G*’) and loss (*G*’’) moduli. 

### 2.4. Thermogravimetric Analysis (TGA)

Thermogravimetric measurements were carried out on a TGA 851e from Mettler-Toledo. Crude humins and humins/pTSA were measured at a heating rate of 2 °C·min^−1^ under air flow (50 mL·min^−1^) from 30 to 300 °C, to simulate the same conditions used during rheology measurements.

### 2.5. Theoretical Calculation

Curing reactions are usually followed by a heat release. The degree of curing is assumed to be directly proportional to the released heat [31]:

(1) $$\alpha_{t}=\frac{\int_{t_{i}}^{t}\left(dQ/dt\right)\;dt}{\int_{t_{i}}^{t_{f}}\left(dQ/dt\right)\;dt}$$

where d*Q*/d*t* corresponds to the heat flow, *t*_i_ is the time at which the process initiates and *t_f_* is the time at which the process finishes. The term $\int_{t_{i}}^{t_{f}}\left(\frac{dQ}{dt}\right)dt$ represents the total reaction heat released during the process. Thus, the reaction rate can be easily studied by DSC. However, kinetic studies can be achieved by linking any chemical or physical property that varies during the reaction with the extent of reaction, and by normalizing the quantity between 0 and 1. For example, if the storage modulus or the heat capacity changes with the reaction progress, an extent of conversion can be defined as follows:

(2) $$\alpha_{t}=\frac{C_{t}-C_{t_{i}}}{C_{t_{f}}-C_{t_{i}}}$$

where *C* represents the physical property measured at time *t*. The general form of the reaction rate is defined as [32]:

(3) $$\frac{d\alpha }{dt}=k\left(T\right)\;f\left(\alpha \right)$$

where $\frac{d\alpha }{dt}$ is the reaction rate, α is the extent of conversion, *k*(*T*) is the rate constant, *t* is the time, *T* is the temperature and *f*(α) is the mathematical function that represents the reaction mechanism. The explicit temperature dependence of the rate constant is obtained by replacing *k*(*T*) with the Arrhenius equation as follows:

(4) $$\frac{d\alpha }{dt}=A\exp\left(-\frac{E}{RT}\right)\;f\left(\alpha \right)$$

where *A* is the pre-exponential factor, *E* is the activation energy, and *R* is the gas constant. This type of analysis does not allow for a possible change in the rate-limiting step [32]. The reaction rate is generally determined by the rate of both chemical reaction and diffusion [33]. The rate limiting process is determined by the ratio of the characteristic times of chemical reaction and diffusion. Viscosity has been demonstrated to play a crucial role in diffusion control [32]. The relaxation time for a molecule in a viscous medium is directly proportional to the viscosity by [34]:

(5) $$\tau =\frac{4\pi a^{3}\eta }{k_{b}T}$$

where *τ* is the relaxation time, *a* is the molecular radius, *η* is the viscosity of the medium, *k_b_* is the Boltzmann’s constant. Thus, an adequate description of the diffusion regime requires the diffusion rate constant to account for a change in the activation energy of diffusion *E_D_* with the progress of crosslinking [32,35,36], as follow:

(6) $$k_{D}\left(T,\alpha \right)=D_{0}\exp(-E_{D}/RT+K\alpha )$$

where *E_D_* is the activation energy of diffusion, *D*_0_ is the pre-exponential factor and *K* is a constant accounting for a change in the activation energy of diffusion with the progress of crosslinking. 

In the case of chemical reactions controlled by diffusion of oligomers, the variation of the effective activation energy *E_α_* can be rationalized in terms of a kinetic model for a process occurring in a mixed diffusion-kinetic regime [37]. The total effective reaction time is the sum of the times of the reaction act and diffusion. Replacing the times with the reciprocal rate constants yields:

(7) $$k_{ef}^{-1}\left(T\right)=k_{}^{-1}\left(T\right)+k_{D}^{-1}\left(T,\alpha \right)$$

where *k*_ef_(*T*), *k*(*T*), and *k*_D_(*T*,α) are respectively the effective rate constant, the reaction rate constant and the diffusion rate constant. The usual Arrhenius expression was used for the rate constant of the chemically controlled part of the reaction:

(8) k(T)=A exp(−E/RT)

where *A* is the pre-exponential factor and *E* is the activation energy of the chemical reaction. According to this model, the experimentally measured effective activation energy, *E_α_* is a function of the activation energies of both chemical reaction and diffusion. Applying the isoconversional principle to Equation (7) lead to [32]:

(9) $$E_{\alpha }=-R{\left(\frac{d\ln k_{ef}}{dT^{-1}}\right)}_{\alpha }=\frac{k\left(T\right)\;E_{D}+k_{D}\left(T,\alpha \right)\;E}{k\left(T\right)+k_{D}\left(T,\alpha \right)}$$

The latter equation suggests that *E_α_* would vary between *E* and *E_D_*. 

### 2.6. Isoconversional Method

Isoconversional methods can detect changes in the rate limiting steps of the overall cure rate. For this reason, isoconversional methods have largely been used for studying the curing kinetics of numerous thermoset resins [35,38,39,40,41]. The isoconversional principle states that the reaction rate at a constant extent of conversion is only a function of temperature. Thus, it is possible to compute a value of *E_α_* for each value of *α* without any assumption on the reaction model. If *E_α_* does not change with *α* it can be concluded that the overall reaction mechanism follows a single-step process. In this case, evaluation of kinetic parameters is an easy task. However, most reactions and specially those occurring with high viscosity variations do not obey this simple kinetic scheme. Significant variations in *E_α_* with *α* indicates a kinetically complex process (i.e., multi-steps) and *E_α_* variations can be approximated to a change in the reaction mechanism [42,43]. It has already been shown that the use of an isoconversional method leads to meaningful mechanistic and kinetic evaluations by analysis of the *E_α_* dependences [44,45,46]. Isoconversional computational methods can generally be split in differential and integral. The Friedman method [47] is the most used differential isoconversional method. Differential methods do not make use of any approximation. However, the practical use of this latter is associated with numerical instabilities [48]. Common integral methods make use of different approximation methods to solve the temperature integral. To achieve greater accuracy, numerical integrations should be used. For this reason, Sbirrazzuoli and Vyazovkin developed the so-called “Ozawa corrected” and the “advanced isoconversional” methods [29,31,49,50]. The advantage of these methods is that they use a numerical integration of the data. In addition, the “advanced isoconversional method” can be applied to any temperature program. In this study, the advanced isoconversional method was used. For a series of *n* experiments performed with different heating programs *T_i_(t)*, the effective activation energy (*E_α_*) can be determined at any extent of conversion *α* by finding the value of *E_α_* that minimized the function:

(10) $$\Phi \left(E_{\alpha }\right)\;=\overset{n}{\underset{i=1}{\sum }}\overset{n}{\underset{j\neq i}{\sum }}\frac{J\left[E_{\alpha },T_{i}\left(t_{\alpha }\right)\right]}{J\left[E_{\alpha },T_{j}\left(t_{\alpha }\right)\right]}$$

In Equation (10), the integral:

(11) $$J\left[E_{\alpha },\;T\left(t_{\alpha }\right)\right]\equiv \int_{t_{\alpha -\Delta \alpha }}^{t_{\alpha }}\exp\left[\frac{-E_{\alpha }}{RT\left(t\right)}\right]\;dt$$

is evaluated numerically for a set of experimental heating program. Integration is performed over small time segments, to eliminate systematic errors. The integral *J* in Equation (10) is evaluated numerically by using the trapezoid rule. *E_α_* is computed for each value of *α* between 0.02 and 0.98 with a step of 0.02. The advantage of using the advanced isoconversional method is the possibility to use the same computational algorithm to evaluate *E_α_* dependence from both isothermal and non-isothermal data. 

## 3. Results and Discussion

### 3.1. Isothermal Investigation

#### 3.1.1. Analysis of the Cure Kinetics by Isothermal DSC Measurements

**Figure 1:**
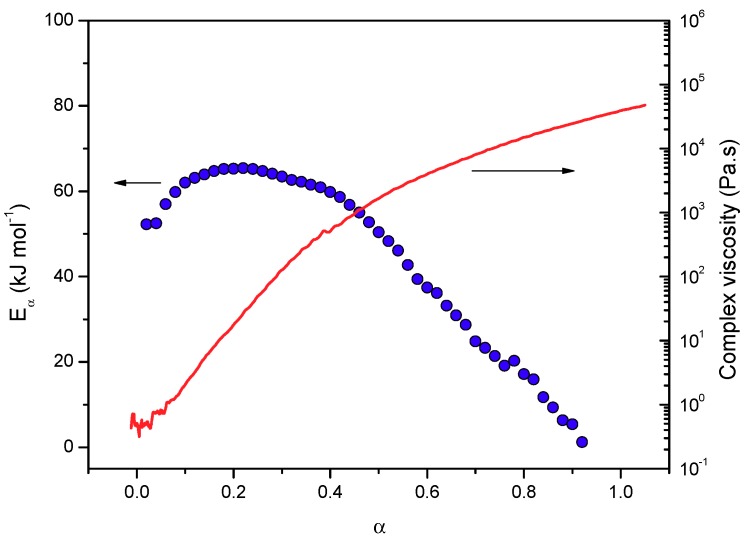
Variation of the effective activation energy (*E*_α_) with the extent of conversion (α) obtained under isothermal conditions (blue circle) and the evolution of the complex viscosity (solid red line).

The curing reaction was first studied by isothermal DSC. The obtained data were used to compute the *E*_α_-dependency, according to Equations (10) and (11). The results are shown in Figure 1, and relate the viscosity changes at 140 °C with the extent of conversion (*α*). Three main steps can be identified to describe the curing reaction. The first step, corresponding to very small degree of conversion, is characterized by an increase of *E*_α_ from 50 kJ·mol^−1^ to around 65 kJ·mol^−1^. The mixture is in the liquid state, with an initial low viscosity (from 10^−1^ to 10^1^ Pa·s). This step is associated with the formation of active species that initiate polymerization. In the second step, from *α* = 0.2 to 0.4, *E*_α_ is almost constant, with a value close to 60 kJ·mol^−1^, indicating that the overall reaction rate is determined by a single step mechanism [45,51]. Humins reach gelation at around α = 0.4 and from this stage the increase of viscosity is slowed down (Figure 1). The high viscosity is now hindering chemical reactions due to restriction of molecular mobility. At this stage of the reaction, the polymerization starts to shift from chemical to diffusion control. For α > 0.4, *E*_α_ is characterized by a sharp decrease to 10 kJ·mol^−1^. This tendency and the low values of *E*_α_ are associated with diffusion controlled mechanism [32,35,38,45]. In this step, the curing process is driven by the mobility of unreacted molecular segments with short range motion. Similar behavior was observed for curing of other biobased resins [27,39].

#### 3.1.2. Analysis of the Cross-Linking Kinetics by Stochastically Temperature Modulated DSC Measurements

**Figure 2:**
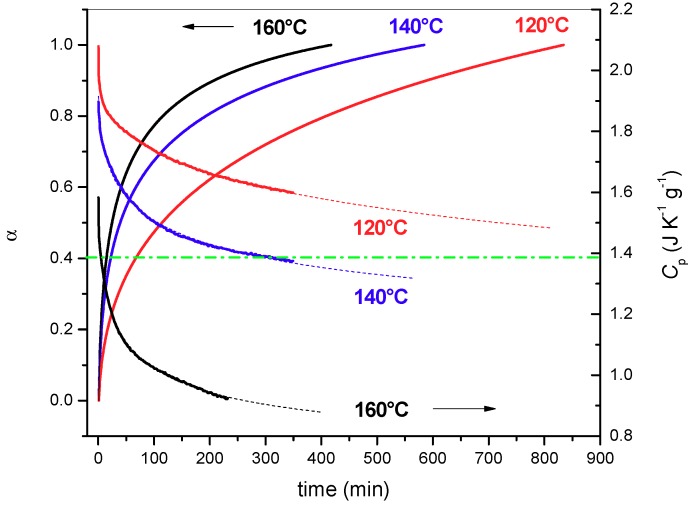
Left: Extent of conversion (*α*) obtained after the application of Equation (2) as a function of time. Right: Topem^®^ curves of *c_p_* during isothermal curing at 120, 140, and 160 °C (solid lines), and fitting data with the KWW relaxation function (dot lines).

Stochastically temperature modulated DSC (Topem^®^) was used for a comparison with the DSC results previously obtained. This is a unique technique, which permits the evaluation of the quasi-static specific heat capacity, *c*_p_, during curing. Figure 2 shows the *c_p_* variation with time, at 120, 140 and 160 °C. Heat capacity sharply decreases at the beginning of the measurements, and then slowly approaches a constant value. During thermal treatment, chain mobility is limited due to a fast increase of the molecular weight during curing reactions, corresponding to a deep decrease in the heat capacity [11,51]. At this temperature, reactions start very fast. Due to the necessary stabilization time of the instrument (1–5 s) the very first seconds are not measured, which explains the differences in the initial values of *c_p_*. These results are in perfect agreement with the previous *E*_α_-dependency variations computed from isothermal DSC heat flux measurements (Figure 1). The initial stages of the curing are controlled by fast chemical reactions followed by a transition to diffusion control (*α* > 0.4) when the mobility of the reaction medium decreases due to the molecular weight increase. This decrease in mobility results in a high decrease of the specific heat capacity as measured with Topem^®^.

The relaxation behavior of curing with time was evaluated by fitting the experimental data with the Kohlrausch-Williams-Watts (KWW) relaxation function [52,53]. This function is used to describe various types of relaxation data. It has been first used for studying dielectric relaxation but nowadays it is applied as a universal tool for studying various physical and chemical processes [54]. The KWW function is expressed as:

(12) $$\Phi_{t}=cste+exp{\left(\frac{-t}{\tau_{1}}\right)}^{\beta_{1}}$$

where $\Phi_{t}$ is the relaxation function, indicating the kinetics of non-equilibrium to equilibrium transformation of the system, *τ* is the mean molecular relaxation time, *cste* is a shift factor and *β* is the relaxation distribution parameter, indicating the deviation of the relaxation from exponential behavior [55]. The KWW function described well the relaxation behavior for the three curves. The fitting was done considering two relaxation times: *τ*_1_ corresponding to the relaxation time of mobile chains in the chemically controlled part of the reaction and *τ*_2_ related to the relaxation of the small molecules in the latter stage of the reaction, corresponding to diffusion kinetics:

(13) $$\Phi_{t}=cste+exp{\left(\frac{-t}{\tau_{1}}\right)}^{\beta_{1}}+exp{\left(\frac{-t}{\tau_{2}}\right)}^{\beta_{2}}$$

The value of *τ* and *β* are shown in Table 1. The lower is the curing temperature, the larger the corresponding *τ*_1_. A significant difference is observed between measurements at 120 and 140/160 °C. The larger *τ*_1_ is attributed to the insufficient energy provided to the system at low temperatures, resulting in a restriction of its relaxation. The three samples show a comparable *τ*_2_, indicating a similar and very slow relaxation behavior of the small molecules in the diffusion controlled part of the reaction (*α* > 0.4). Values of *β* are much smaller than 1, indicating a non-exponential and broad distribution of the *c_p_* relaxations [56].

**Figure 3:**
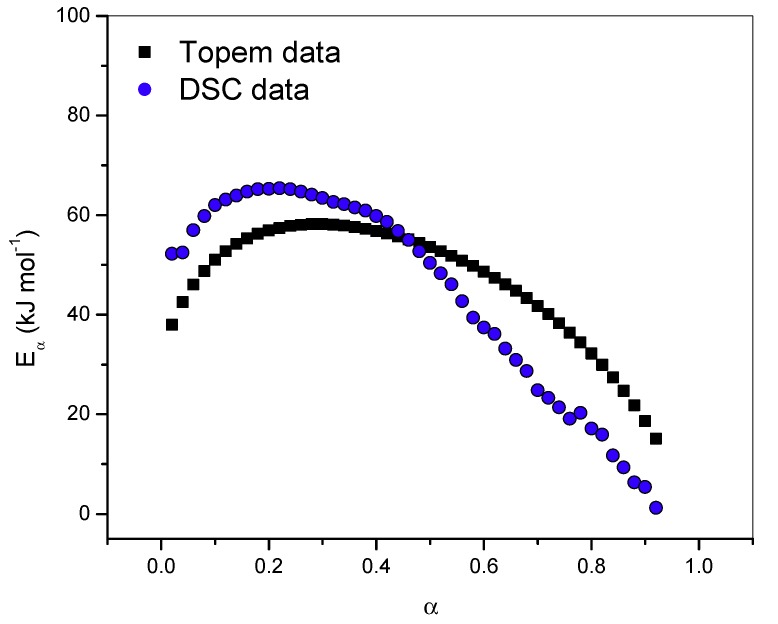
Evolution of the effective activation energy (*E*_α_) as a function of the extent of conversion (*α*) after treatment of DSC data (blue dots) and Topem^®^ data (black squares) obtained by isothermal curing.

To compute the *E*_α_-dependency from Topem^®^ data, the heat capacity (*c_p_*) was normalized between 0 and 1 according to Equation (2) and an extent of conversion *α* was obtained for each temperature (Figure 2). The advanced isoconversional method previously described was used to compute these data, according to Equations (10) and (11). The *E*_α_-dependency is shown in Figure 3 and compared with the results obtained by standard DSC. The results obtained with the two techniques fit well, indicating the same tendencies. The same kinetics steps can be identified, and the predominance of a diffusion kinetic mechanism for *α* > 0.4 is confirmed. The consistency of this result is remarkable. Despite in isothermal DSC the heat flux is treated while in Topem^®^ the *c_p_* is treated, both results lead to the same conclusions.

#### 3.1.3. Relation between Extent of Cure (*α*) and Glass Transition Temperature (*T_g_*)

**Figure 4:**
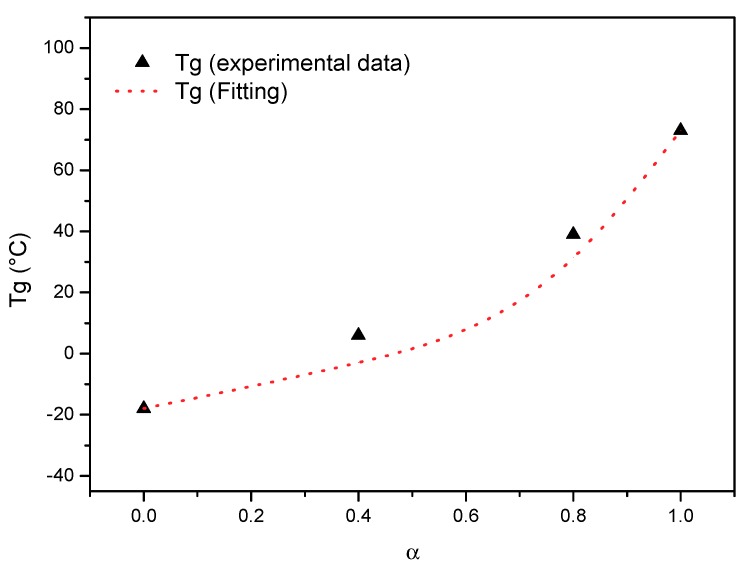
*T*_g_ evaluation at different degrees of conversion (black triangles) and fitting with the Di Benedetto equation (dash red line).

Optimum curing is difficult to achieve in thermosetting systems because of the high changes in the viscosity during the crosslinking of the material. As previously noticed, the reaction may start to be controlled by diffusion at the early stages of the reaction resulting in incomplete cure and loss of mechanical properties. Establishing a relation between the *T_g_* and the extent of conversion (*α*) is of major importance for the optimization of the final properties of the material. The glass transition temperature can be correlated to the extent of conversion according to the Di Benedetto equation [57,58,59]:

(14) $$\frac{T_{g}-T_{g0}}{T_{g\infty }-T_{g0}}=\frac{\lambda \alpha }{\left[1-\left(1-\lambda \right)\alpha \right]}$$

with *λ* given by:

(15) $$\lambda =\frac{\Delta c_{p\infty }}{\Delta c_{p0}}$$

where $T_{g0}$ is the value for *α* = 0, corresponding to the *T_g_* of the unreacted system, $T_{g\infty }$ is the “infinite” *T_g_* corresponding to the maximum value that can be obtained, ideally the value for *α* = 1 and *λ* is a fitting parameter, which was found to be equal to 0.30 by the nonlinear fitting. This value is close to what was previously observed for other thermosetting systems [60]. This parameter was obtained by DSC measurement from the ratio of Δ*c_p_* of the glass transition of the fully cured system (Δ*C_p_*_∞_) divided by the Δ*c_p_* of the glass transition of uncured system (Δ*c_p_*_0_). Figure 4 shows the *T*_g_ evaluation at different extent of conversion obtained from experimental data, and the fitting values obtained with the Di Benedetto equation. The equation seems to fit quite well the experimental data, suggesting that it can be used to extrapolate value of *T*_g_ at every needed *α*. In this system, the value of *λ* is found to be 0.34, which is very close to the value obtained by nonlinear fitting (0.30).

### 3.2. Non-Isothermal Investigation

**Figure 5:**
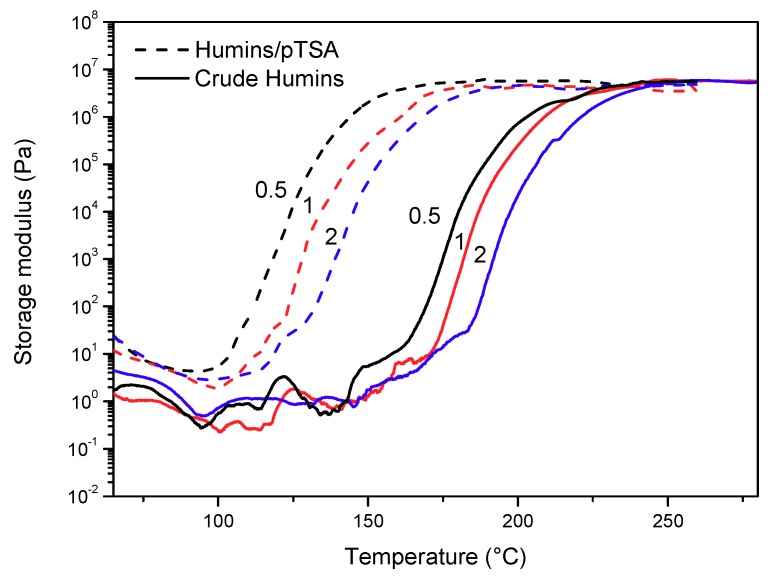
Storage modulus (*G*’) variation with the temperature at different heating rates (0.5, 1 and 2 °C·min^−1^), for crude humins (solid lines) and humins/pTSA (dash lines).

Due to the high complexity and heterogeneity of humins, it was not possible to properly treat the signal from DSC data in non-isothermal mode. Thus, isoconversional analysis has been applied to the rheological studies of humins cross-linking in non-isothermal conditions at 0.5, 1 and 2 °C·min^−1^. The storage modulus (*G*’) variation with temperature was measured for crude humins and for humins/pTSA samples (Figure 5). A sharp increase of the modulus is identified at the beginning of cross-linking due to the polymer chain growth, and thus to molecular weight increase. The starting temperature of the curing process is higher when increasing the heating rate, which is a phenomenon widely described in the literature [27]. Significant differences are observed between the behavior of crude humins and humins/pTSA. In the case of crude humins, a first plateau is identified from around 100 to 150 °C. In this range of temperatures, the competition between the temperature-dependence of viscosity and the increase of molecular weight results in a quasi-constant value of *G*’. A sharp increase in *G*’ is observed from about 150 °C and the final plateau, corresponding with end of curing which is reached at around 240 °C. When pTSA is added, *G*’ reaches a minimum around 100 °C and then sharply increases up to 180 °C, reaching a plateau. The use of an accelerator allows cross-linking reactions to be initiated very fast, and no plateau is observed anymore at low temperatures. All the curves are shifted toward much lower temperatures in case of humins/pTSA, which confirms the strong “catalytic” effect of pTSA on humins’ crosslinking.

**Figure 6:**
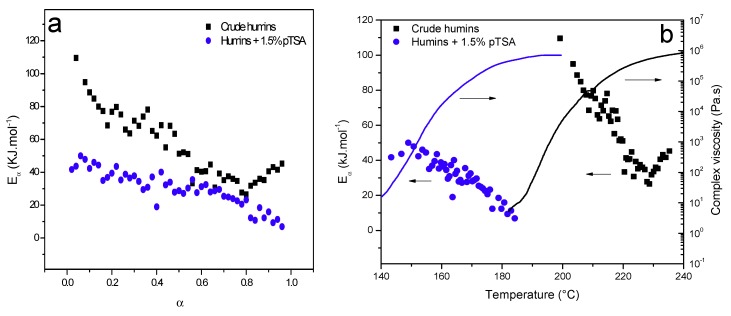
Evolution of the effective activation energy (*E*_α_) as function of (**a**) the extent of conversion (*α*) and (**b**) the temperature, evaluated from non-isothermal data for crude humins (black squares) and Humins/pTSA (blue circles) and (**b**) complex viscosity and effective activation energy (*E*_α_) variation with temperature.

*G*’ was normalized between 0 and 1 according to Equation (2) and an extent of conversion *α* related to rheological behaviour was obtained. The advanced isoconversional method was used to compute the obtained data, from Equations (10) and (11). Figure 6 shows the resulting *E*_α_-dependency as a function of *α* (Figure 6a) and temperature (Figure 6b). In Figure 6b, this dependency is compared with the variation of viscosity. As previously observed in the isothermal investigations, the *E*_α_-dependency shows complex variations that are associated with a complex cure mechanism involving several stages. Crude humins sample shows a first steep decrease of *E*_α_ for *α* < 0.35 that can be attributed to an autocatalytic step [32,43]. The first value of 110 kJ·mol^−1^ represents the activation energy for non-catalyzed curing of humins [61]. During this stage, *E*_α_ goes from 110 to 65 kJ·mol^−1^. A second step is identified from *α* > 0.35. As observed in Figure 6b, at this stage the viscosity has strongly increased, hindering molecular mobility. The overall polymerization is now controlled by the motion of short chains. Thus, diffusion is the rate limiting process. During this step, *E*_α_ smoothly decreases to 25 kJ·mol^−1^, which is in good agreement with a diffusion control at the end of the reaction [51]. A final step is then observed for *α* > 0.8, characterized by a slow increase of *E*_α_. The high temperature reached might increase molecular mobility, promoting chemical reaction to be reactivated. This may correspond to motions of longer chain segments [44,45].

A similar dependency is observed for humins/pTSA. The curing processes of the two systems have the same initiation steps and slightly different mechanisms of propagation. As observed in the case of crude humins, a first step is identified up to *α* = 0.35, when *E*_α_ slightly decreases from around 45 to around 32 kJ·mol^−1^. The cross-linking is catalyzed by pTSA thus the initiation reactions occur at significantly lower temperatures and with a lower value of *E*_α_. Moreover, the slope of the viscosity curve in this first step is also significantly lower. A second step is observed from *α* = 0.35 to *α* = 0.60, associated to an almost constant value of energy. This indicates that this second step is controlled by a single step. Finally, *E*_α_ decreases from ~ 32 to 7 kJ·mol^−1^. As previously explained, this is typical of a diffusion control mechanism that occurs at high temperatures due to the increase of viscosity, as observed in Figure 6b, and cross-linking is controlled by the motion of short chains. Similar values of activation energies have been reported for epoxy-amine [51], PF resins [62] and FA resins [27].

**Figure 7:**
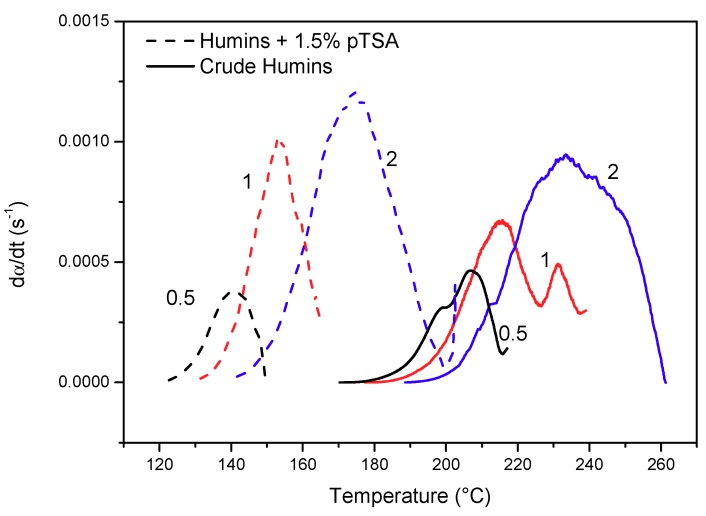
Reaction rate d*α*/d*t* versus temperature of crude humins (solid lines) and humins/pTSA (dash lines) at different heating rates. The heating rate of each experiment (in °C·min^−1^) is indicated by each curve.

It is of interest also to compare the reaction rate (d*α*/d*t*) of the two samples at different heating rates. The relationship between the rheological conversion rate, d*α*/d*t*, and the temperature is shown in Figure 7, revealing the effect on accelerator on the cross-linking rate. The maximum rate achieved varied according to the heating rate used. The reaction rates are higher for humins/pTSA when heating rates of 1 and 2 °C·min^−1^ are used. The use of accelerator is not only decreasing the initiation temperature, but it also contributes to increasing the reaction rate. This is not valid for heating rate of 0.5 °C·min^−1^, probably because the initiation temperature is considerably low and thus reaction rate is slowed down. A shoulder is observed for crude humins, indicating that two different curing mechanisms are initiated at different temperatures. This agrees with what was observed in Figure 6. Similar tendencies have been reported for other thermosetting resin [63].

**Figure 8:**
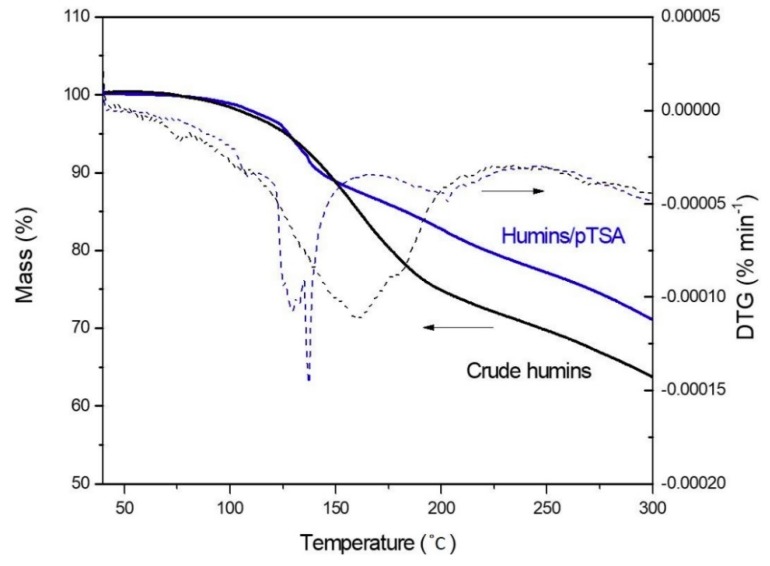
TG and DTG measurement of crude humins and humins/pTSA, heating rate 2 °C min^−1^ under air.

The mass loss of crude humins and humins/pTSA during cross-linking was studied under air, to simulate the same conditions as the rheological measurements (Figure 8). The same behavior is observed up to 140 °C, where a mass loss of 10% is reached. For higher temperatures, crude humins show a higher mass loss, showing a deviation up to 10% with humins/pTSA. This difference might be linked to the different curing mechanisms previously reported. Indeed, as already observed in Sangregorio et al. [11], this mass loss is linked to volatiles and small molecules which are released, together with water from polycondensation products, during cross-linking. When using an accelerator, cross-linking reactions are initiated at lower temperature (Figure 5), thus small molecules are most likely involved in the reaction before starting volatilization. For this reason, the mass loss is not observed in TGA measurements.

## 4. Conclusions

The kinetics of humins cross-linking by advanced isoconversional analysis have been proposed for isothermal and non-isothermal data. For this latter, both the curing of crude humins and humins with an accelerator were studied and compared. Due to the complexity of the material, different techniques have been used for these purposes. Variation of the effective activation energy with conversion was obtained and different curing mechanisms were proposed. Isothermal data were studied first by standard DSC and Topem^®^. We demonstrated consistency in results obtained by treating the heat flow signal from DSC and the quasi-static heat capacity (*c_p_*_0_) signal from Topem^®^. Storage modulus variation was then used to study the curing mechanism under non-isothermal mode. Consistent results from isothermal and non-isothermal data were obtained, giving important correlations with the rheological behaviour during curing. This study leads to the conclusion that the overall humins polymerization follows multi-step kinetics with a predominance of diffusion control mechanism for high degree of conversion (starting from around α = 0.35). The use of pTSA significantly decreases the initiation temperature and the activation energies and increases the curing rate of reaction. Similar mechanisms are identified for crude humins and when using an accelerator. This study brings first insights into humins cross-linking mechanism for optimization of alternative green thermosets.

## Figures and Tables

**Table 1:** Relaxation time (*τ*) and relaxation distribution parameter (*β*) obtained from Equation (13) for isothermal curing at 120, 140 and 160 °C.

*T* (°C)	*τ*_1_ (min)	*τ*_2_ (min)	*β*_1_	*β*_2_
120	1.58 × 10^3^	7.10 × 10^7^	3.96 × 10^−1^	2.26 × 10^−4^
140	1.25 × 10^2^	7.40 × 10^7^	3.10 × 10^−1^	1.25 × 10^−4^
160	3.93 × 10^1^	7.40 × 10^7^	3.52 × 10^−1^	1.24 × 10^−4^
